# TBC1D15-regulated mitochondria–lysosome membrane contact exerts neuroprotective effects by alleviating mitochondrial calcium overload in seizure

**DOI:** 10.1038/s41598-024-74388-3

**Published:** 2024-10-10

**Authors:** Yinyin Xie, Wanwan Zhang, Tingting Peng, Xiaoyi Wang, Xiaolei Lian, Jiao He, Cui Wang, Nanchang Xie

**Affiliations:** 1 Department of Neurology, The First Affiliated Hospital of Zhengzhou University, Zhengzhou, 450052 China; 2 Institutes of Biological and Medical Sciences, Suzhou Medical College of Soochow University, Suzhou, 215123 China; 3 Department of Clinical Laboratory, Key Clinical Laboratory of Henan Province, The First Affiliated Hospital of Zhengzhou University, Zhengzhou, 450052 China

**Keywords:** Seizure, TBC1D15, Mitochondria–lysosome membrane contact, Mitochondrial calcium overload, Cellular neuroscience, Diseases of the nervous system

## Abstract

Mitochondrial calcium overload plays an important role in the neurological insults in seizure. The Rab7 GTPase-activating protein, Tre-2/Bub2/Cdc16 domain family member 15 (TBC1D15), is involved in the regulation of mitochondrial calcium dynamics by mediating mitochondria–lysosome membrane contact. However, whether TBC1D15-regulated mitochondria–lysosome membrane contact and mitochondrial calcium participate in neuronal injury in seizure is unclear. We aimed to investigate the effect of TBC1D15-regulated mitochondria–lysosome membrane contact on epileptiform discharge-induced neuronal damage and further explore the underlying mechanism. Lentiviral vectors (Lv) infection and stereotaxic adeno-associated virus (AAV) injection were used to regulate TBC1D15 expression before establishing in vitro epileptiform discharge and in vivo status epilepticus (SE) models. TBC1D15’s effect on inter-organellar interactions, mitochondrial calcium levels and neuronal injury in seizure was evaluated. The results showed that abnormalities in mitochondria–lysosome membrane contact, mitochondrial calcium overload, mitochondrial dysfunction, increased levels of reactive oxygen species, and prominent neuronal damage were partly relieved by TBC1D15 overexpression, whereas TBC1D15 knockdown markedly deteriorated these phenomena. Further examination revealed that epileptiform discharge-induced mitochondrial calcium overload in primary hippocampal neurons was closely associated with abnormal mitochondria–lysosome membrane contact. This study highlights the crucial role played by TBC1D15-regulated mitochondria–lysosome membrane contact in epileptiform discharge-induced neuronal injury by alleviating mitochondrial calcium overload.

**Supplementary Information:**

The online version contains supplementary material available at 10.1038/s41598-024-74388-3.

## Introduction

Among the numerous possible mechanisms underlying epileptogenesis, seizures, and epilepsy, the mitochondria have attracted considerable attention in recent years^1,2^. They are essential intracellular organelles responsible for generating the main source of cellular energy, adenosine triphosphate (ATP), by coupling oxidative phosphorylation with respiration^3^. Seizure-induced cell death is linked to mitochondrial dysregulation, characterized by a depolarized mitochondrial membrane potential, reduced ATP production, increased reactive oxygen species (ROS) release, impaired calcium homeostasis, and leakage of pro-apoptotic factors into the cytoplasm, such as cytochrome c, which have detrimental effects on cell function and fate^4^. Mitochondria have been in the spotlight in seizure-induced cell death. Therefore, the maintenance of mitochondrial homeostasis is crucial for the physiological function and survival of cells.

Mitochondrial calcium maintains mitochondrial homeostasis by regulating mitochondrial dynamics, mitophagy, and mitochondrial biogenesis^1,5,6^. Impaired mitochondrial calcium homeostasis leads to compromised mitochondrial quality control, contributing to several pathological conditions, such as seizure^1^. The mitochondrial calcium uniporter (MCU) complex in the inner mitochondrial membrane (IMM) is the main site of calcium ion entry into the mitochondria^7^. MCU mediates mitochondrial calcium uptake during seizures; MCU inhibition can reduce ROS production and thus combat seizure-induced neuronal damage^8,9^. The opening of the mitochondrial permeability transition pore (mPTP) located in the IMM is an alternative pathway for calcium ion transport across the mitochondrial membrane^1^. Mitochondrial calcium overload can trigger mPTP opening, resulting in the loss of mitochondrial membrane potential (MMP); this causes a decrease in ATP synthesis and the release of mitochondrial contents, such as the Bcl2 family proteins, Bax, and cytochrome c, ultimately leading to the induction of apoptosis and cell death^10,11^. mPTP opening mediates cell death, and inhibition of mPTP opening prevents mitochondrial membrane depolarization, which is the initial event in the process of cell death after epileptiform discharge^12^. Therefore, calcium homeostasis in the mitochondria plays a pivotal role in the modulation of cellular physiology and pathophysiology. However, the precise mechanisms underlying the mitochondrial calcium homeostasis imbalance and epileptiform discharge-induced neuronal damage remain unclear.

Interest in studies of inter-organellar communication between mitochondria and various organelles, such as lysosomes, has increased in recent years^13,14^. Distinct from autophagy, mitochondria-derived vesicles, and mitochondria-derived compartments, the direct physical membrane contact between mitochondria and lysosomes, known as mitochondria–lysosome membrane contact, plays a key role in regulating organelle network dynamics and maintaining cellular homeostasis^14–16^. The dynamics of mitochondria-lysosome membrane contact are finely regulated by specific tethering/untethering protein machinery. Active GTP-bound lysosomal Rab7 was found to promotes contact formation, whereas the Rab7 GTPase-activating protein Tre-2/Bub2/Cdc16 domain family member 15 (TBC1D15) mediates contact untethering by driving RAB7 GTP hydrolysis^17,18^. Mitochondria–lysosome membrane contact regulates mitochondrial calcium dynamics through lysosomal main calcium efflux channels, transient receptor potential mucolipin 1 (TRPML1); TRPML1 directly mediates calcium transfer from the lysosomes to the mitochondria at mitochondria–lysosome membrane contact sites^15^. Further investigation revealed that HCT116 TBC1D15 knockout cells showed increased duration of mitochondria–lysosome membrane contact tethering and activation of TRPML1 channels with the agonist ML-SA1 resulted in a increase in total, maximum, and mean mitochondrial calcium, and mitochondrial calcium at multiple time points, compared with wild-type cells^15^. This observation suggests that prolonged contact duration entailed an excessive influx of calcium from lysosomes into the mitochondria, resulting in alterations in mitochondrial calcium levels. However, the precise role of TBC1D15-regulated mitochondria–lysosome membrane contact in seizure remains unclear.

Thus, this study aimed to investigate the effect of TBC1D15-regulated mitochondria–lysosome membrane contact on neuronal damage in the lithium-pilocarpine (PILO) induced status epilepticus (SE) model and the primary hippocampal neuron epileptiform discharge model and further explore the underlying mechanism. Our data indicated that TBC1D15-regulated mitochondria–lysosome membrane contact exerted neuroprotective effects by alleviating mitochondrial calcium overload in seizure.

## Results

### TBC1D15 is overexpressed in vivo status epilepticus and in vitro epileptiform discharge models

**Fig. 1:**
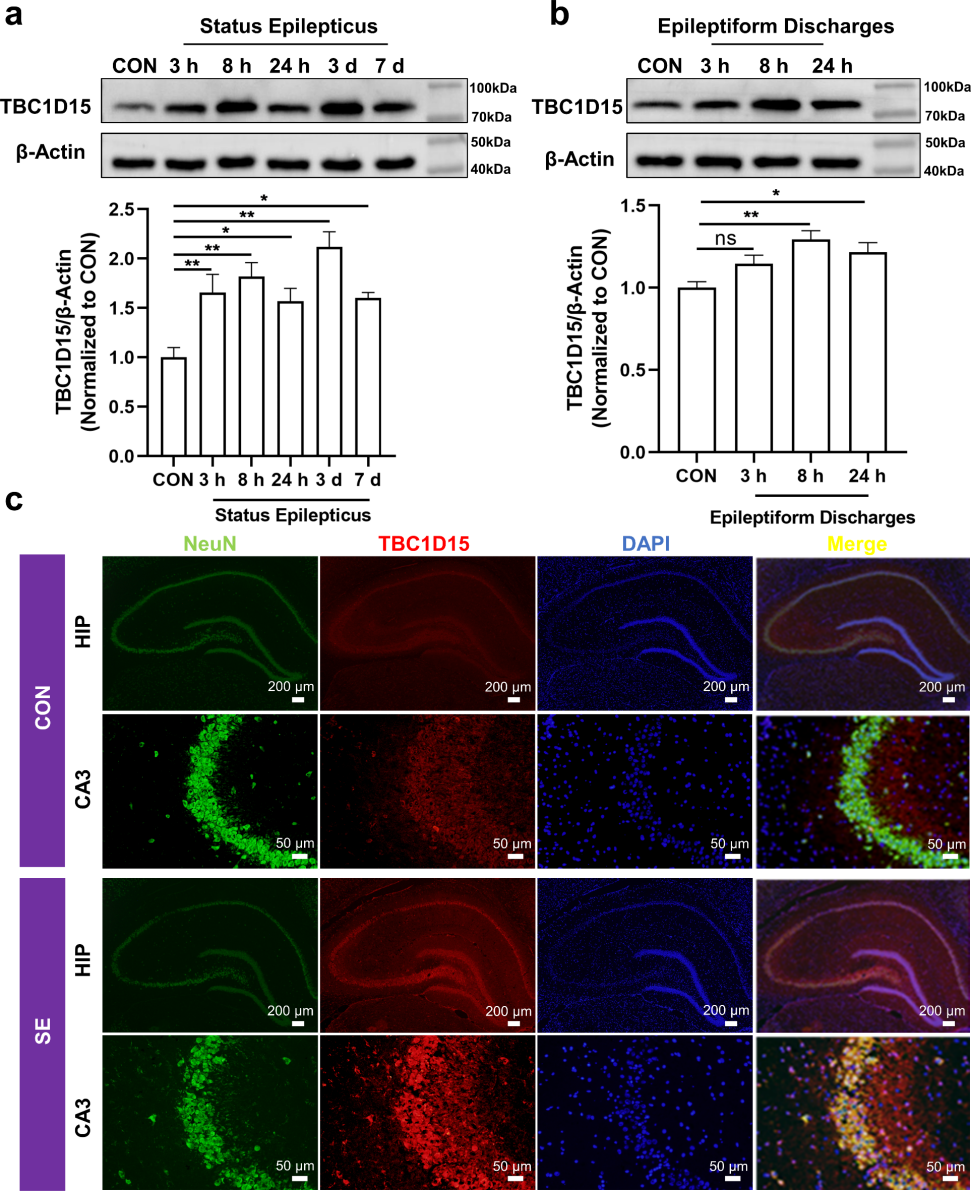
Increased TBC1D15 expression in in vivo SE and in vitro epileptiform discharge models and cellular localization of TBC1D15 in the hippocampal region. (**a** and **b**) TBC1D15 expression variations in vivo and in vitro; (**c**) Double staining of TBC1D15 (red) and NeuN (green) in the hippocampal region of CON and SE rat at 24 h after PILO injection (magnification, ×40 and ×200). ns: not significant, *P* > 0.05; * *P* < 0.05, ** *P* < 0.01, *n* = 5.

TBC1D15 expression in vivo and in vitro was detected by western blotting (WB). In the in vivo model, rat hippocampi were collected at 3 h, 8 h, 24 h, 3 days, and 7 days after modeling. The results revealed that at each time point, TBC1D15 expression was upregulated in the in vivo SE model compared with the control (CON) group (Fig. 1a). Similarly, compared with that in the CON group, the expression of TBC1D15 at all-time points was higher after epileptiform discharge model construction in vitro, although TBC1D15 expression initially increased and then decreased (Fig. 1b). Ultimately, we chose 24 h after modeling, whether in vivo or in vitro, as our time point to assess the corresponding experimental indicators. To analyze the cellular localization of TBC1D15 in the CA3 of the rat hippocampi, we performed double immunofluorescence staining specific for TBC1D15 and NeuN (a neuron marker). The results revealed that TBC1D15 was predominantly localized in neurons within the rat hippocampi (Fig. 1c).

### Effect of lentiviral vectors on neuronal morphology and cell viability

To determine whether lentiviral vectors (Lv) infection affects neuronal morphology, neurons were observed under a microscope 3 days post-infection and before Mg^2+^-free extracellular fluid treatment. It was found that neurons in different groups displayed well-developed neurites (Supplementary Fig. S1a). Quantitative analysis revealed no significant differences in neurite length among neurons infected with negative control Lv (Lv-Nc), TBC1D15 overexpression Lv (Lv-TBC1D15), or TBC1D15 shRNA Lv (Lv-shTBC1D15) compared with the control (CON) group (Supplementary Fig. S1a). Furthermore, gcGFP-based visualization confirmed that neurite length in the Lv-TBC1D15 and Lv-shTBC1D15 groups remained comparable to that in the Lv-Nc group (Supplementary Figure. S1b). Additionally, Cell Counting Kit-8 (CCK-8) assays indicated that cell viability was unaffected by Lv-Nc, Lv-TBC1D15, or Lv-shTBC1D15 infection (Supplementary Fig. S1c).

### Protection exerted by TBC1D15 overexpression against epileptiform discharge-induced neuronal injury

**Fig. 2:**
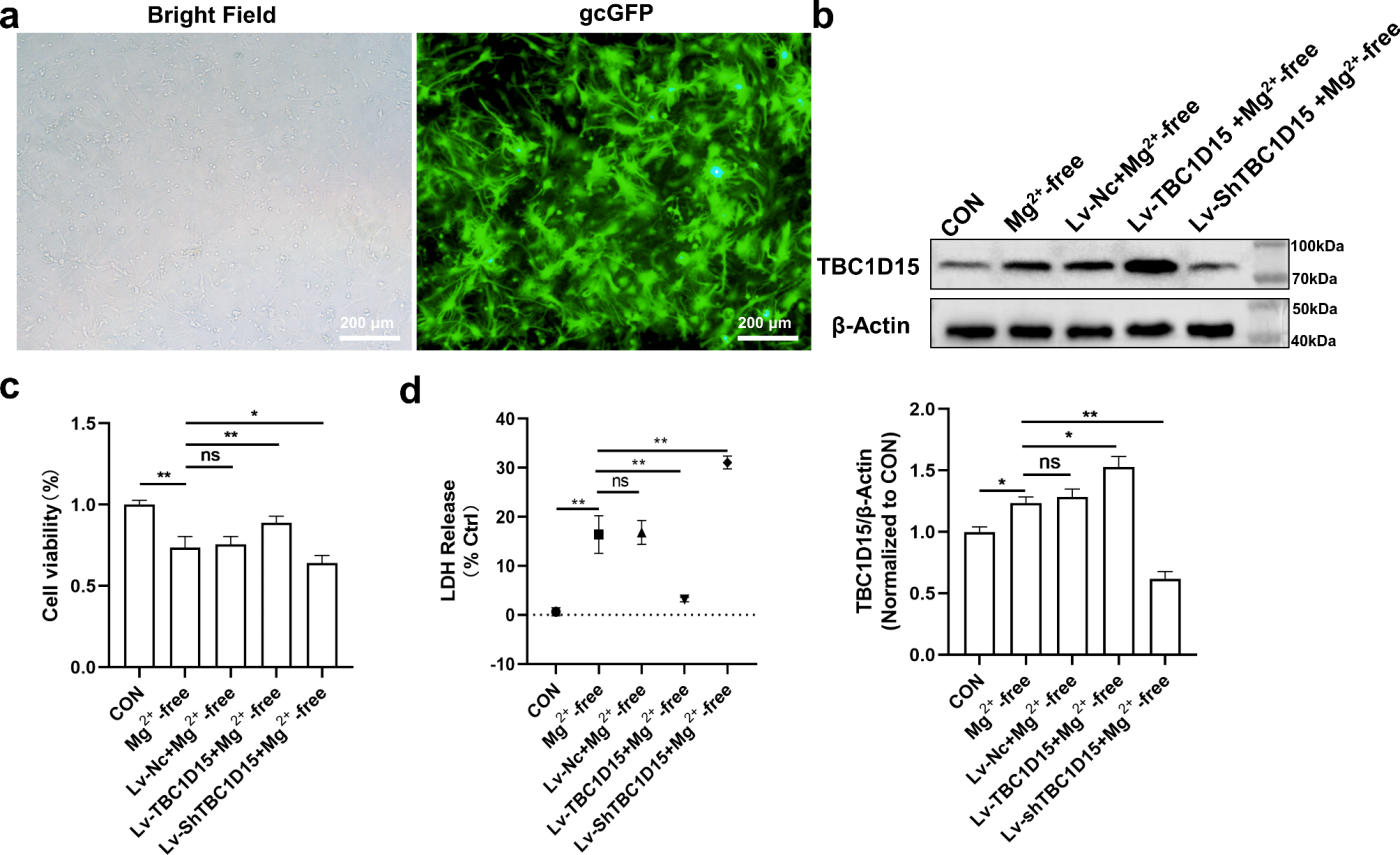
Effect of TBC1D15 on primary hippocampal neurons treated with Mg^2+^-free extracellular fluid. (**a** and **b**) Infection efficiency of Lv was verified by gcGFP fluorescence imaging and WB (magnification, ×100). (**c**) The effect of TBC1D15 on the cell viability of primary hippocampal neurons was detected by the CCK8 assay; (**d**) LDH release assay showed the effects of different TBC1D15 levels on cytotoxicity. ns: not significant, *P* > 0.05; * *P* < 0.05, ** *P* < 0.01, *n* = 5.

To assess the effects of TBC1D15 on cell viability and toxicity mediated by epileptiform discharge in vitro, we utilized CCK-8 and lactate dehydrogenase (LDH) assays, respectively. Initially, gcGFP fluorescence imaging and WB results were used to determine infection efficiency (Fig. 2a and b). CCK-8 assay revealed that neurons in Mg^2+^-free group exhibited a decrease in cell viability compared with the CON group (Fig. 2c). The Lv-TBC1D15 + Mg^2+^-free group’s viability was higher than that of the Mg^2+^-free group (Fig. 2c). Additionally, LDH levels of the Mg^2+^-free group exhibited an increase compared with the CON group, while LDH levels decreased in the Lv-TBC1D15 + Mg^2+^-free group compared with the Mg^2+^-free group (Fig. 2d). However, knockdown of TBC1D15 in the primary hippocampal neurons yielded the opposite results compared with the Mg^2+^-free group in both CCK8 and LDH assays (Fig. 2c and d). There were no significant differences between the Mg^2+^-free and Lv-Nc + Mg^2+^-free groups in CCK8 and LDH assays (Fig. 2c and d).

### Effect of TBC1D15 overexpression on MMP and ROS levels in primary hippocampal neurons of epileptiform discharge model.

**Fig. 3:**
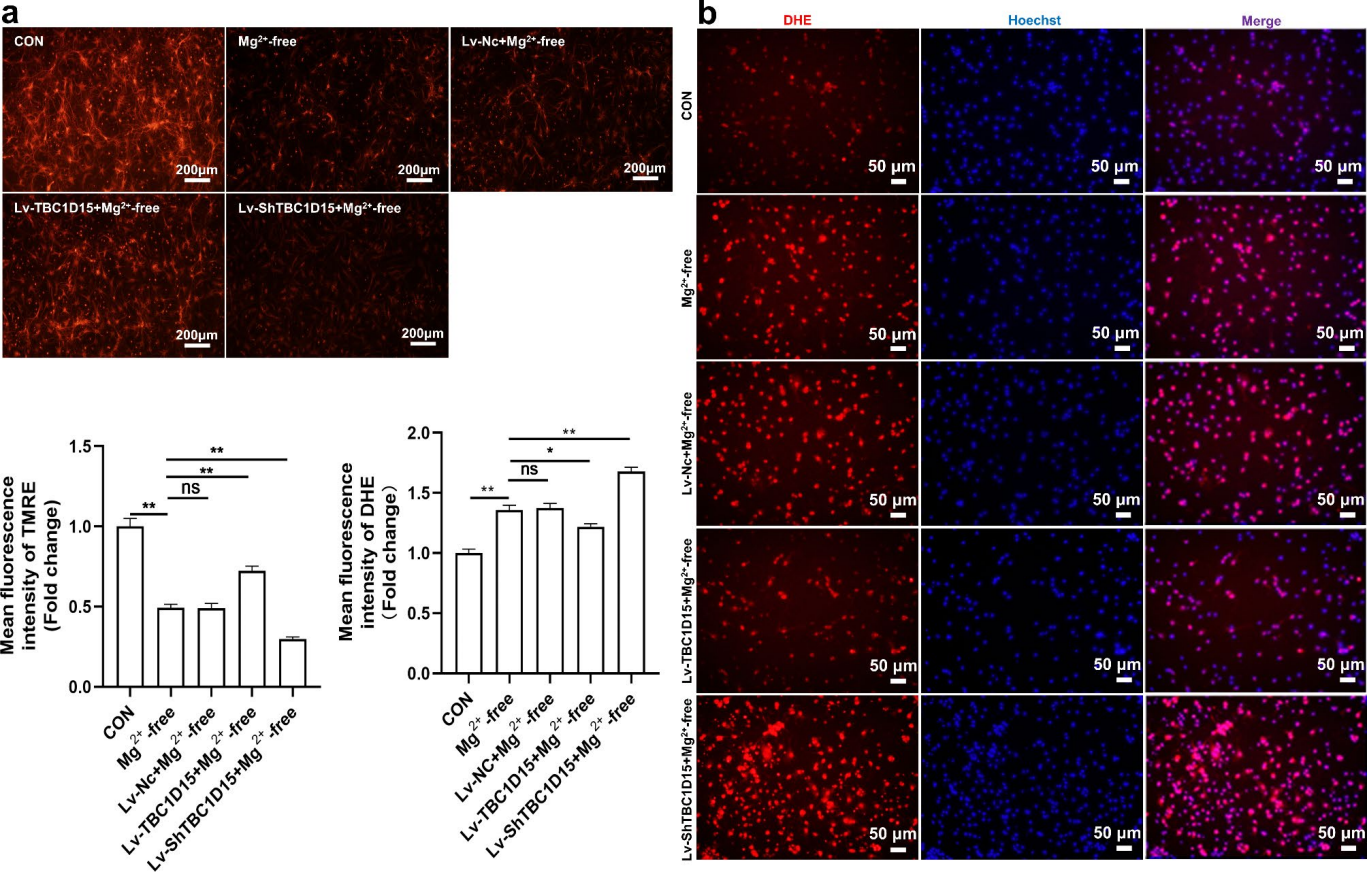
Effect of TBC1D15 on MMP and ROS levels in primary hippocampal neurons treated with Mg^2+^-free extracellular fluid. (**a** and **b**) Representative images and fluorescence quantification of MMP and ROS in different groups (magnification, ×100 and ×200). ns: not significant, *P* > 0.05; * *P* < 0.05, ** *P* < 0.01, *n* = 5.

Primary hippocampal neurons were stained with tetramethyl-rhodamine ethyl ester (TMRE) to assess MMP. In contrast to that in the CON group, the neuronal MMP was decreased in the Mg^2+^-free group. However, TBC1D15 overexpression remarkably increased the MMP, whereas the Lv-shTBC1D15 + Mg^2+^-free group displayed reversed effects of TBC1D15 on the MMP, compared with the Mg^2+^-free group (Fig. 3a). No significant differences were observed between the Mg^2+^-free and Lv-Nc + Mg^2+^-free groups (Fig. 3a). We further evaluated intracellular ROS levels using Dihydroethidium (DHE) fluorescence intensity. The results indicated that the Mg^2+^-free group had a higher DHE fluorescence intensity than that of the CON group (Fig. 3b), implying an increase in ROS levels. The DHE fluorescence intensity decreased and increased in the Lv-TBC1D15 + Mg^2+^-free and Lv-shTBC1D15 + Mg^2+^-free groups compared with the Mg^2+^-free group, respectively (Fig. 3b). No significant differences in ROS levels were observed between the Mg^2+^-free and Lv-Nc + Mg^2+^-free groups (Fig. 3b).

### TBC1D15 regulatory effect on mitochondria–lysosome membrane contact in primary hippocampal neurons of epileptiform discharge model

**Fig. 4:**
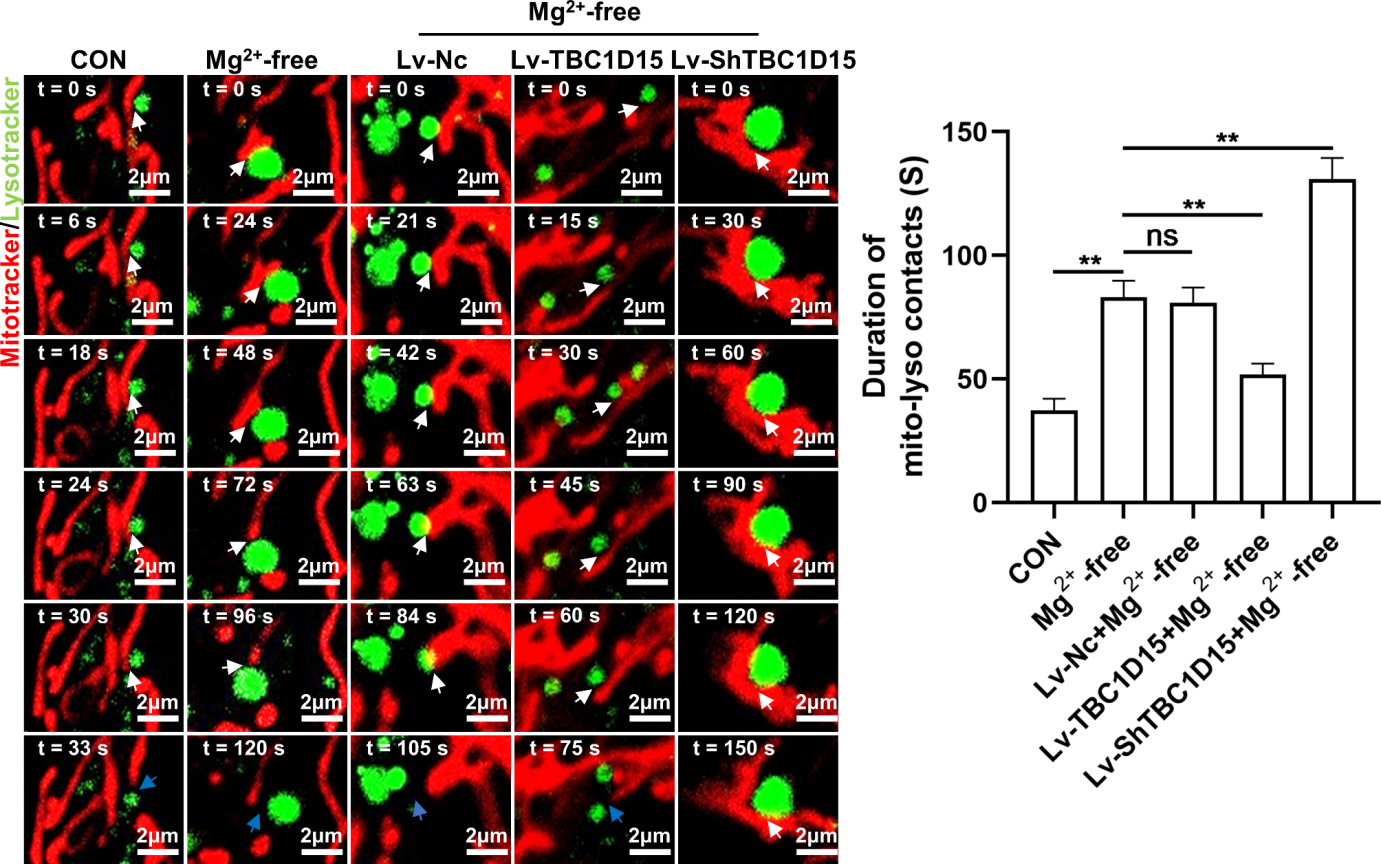
Role of TBC1D15 on mitochondria–lysosome membrane contact. Representative live cell time-lapse confocal images of mitochondria–lysosome membrane contact (white arrows) and contact untethering (blue arrows) were shown (left, magnification, ×1000), and the duration of mitochondria–lysosome membrane contact was analyzed (right), *n* = 3. ns: not significant, *P* > 0.05; * *P* < 0.05, ** *P* < 0.01.

We examined the mitochondria–lysosome membrane contact in primary hippocampal neurons treated with Mg^2+^-free extracellular fluid using time-lapse confocal imaging. In comparison with that in the CON group, the mitochondria–lysosome membrane contact duration was excessively prolonged in the Mg^2+^-free group (Fig. 4). However, the overexpression of TBC1D15 alleviated the increase in mitochondria–lysosome membrane contact duration caused by epileptiform discharges, but the knockdown of TBC1D15 further increased the mitochondria–lysosome membrane contact duration compared with the Mg^2+^-free group (Fig. 4). The Mg^2+^-free and Lv-Nc + Mg^2+^-free groups had similar mitochondria–lysosome membrane contact durations (Fig. 4). These results indicate that epileptiform discharges disrupt the mitochondria–lysosome membrane contact temporal dynamic balance, which is regulated by TBC1D15.

### TBC1D15-regulated mitochondria–lysosome membrane contact may affect mitochondrial calcium homeostasis in primary hippocampal neurons of epileptiform discharge model.

**Fig. 5:**
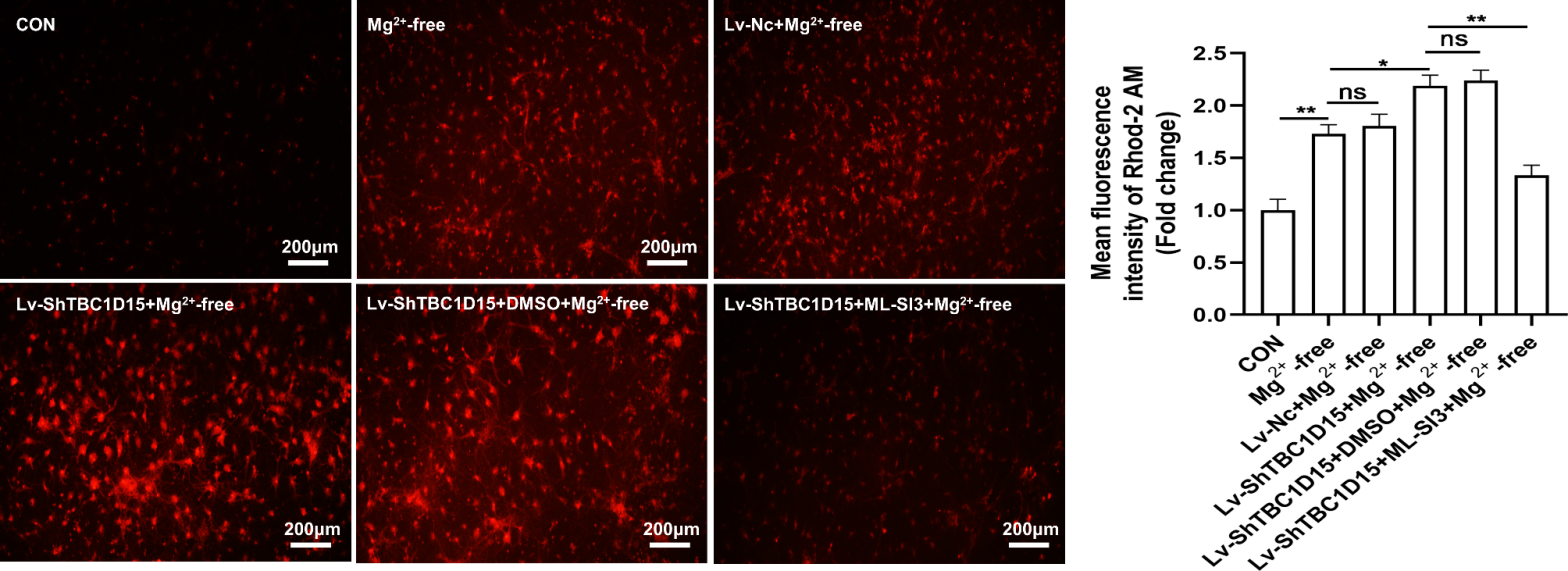
Effect of TBC1D15 on mitochondrial calcium levels. Representative fluorescent images of Rhod-2 AM showed changes in mitochondrial calcium levels in each group (left, magnification, ×100), and the mean fluorescent intensity was quantified (right), *n* = 5. ns: not significant, *P* > 0.05; * *P* < 0.05, ** *P* < 0.01.

To ascertain whether abnormal mitochondria–lysosome membrane contact is associated with mitochondrial calcium overload in the epileptiform discharge model, we assessed mitochondrial calcium levels in primary hippocampal neurons after TBC1D15 knockdown combined with the TRPML1 antagonist ML-SI3 treatment. After the knockdown of TBC1D15 expression, calcium overload was further exacerbated in the primary hippocampal neurons of the epileptiform discharge model (Fig. 5). However, the TBC1D15 knockdown with ML-SI3 reversed this effect (Fig. 5). Interestingly, compared with Mg^2+^-free groups, TBC1D15 overexpression also alleviated neuronal mitochondrial calcium overload induced by epileptiform discharge (Supplementary Fig. S2). Taken together, we hypothesized that untimely mitochondria–lysosome membrane contact untethering/prolonged mitochondria–lysosome membrane contact tethering causes excess mitochondrial calcium uptake from lysosomes, leading to mitochondrial calcium overload, at least in part.

### Upregulation of TBC1D15 suppressed hippocampal neuron apoptosis in primary hippocampal neurons of epileptiform discharge model

**Fig. 6:**
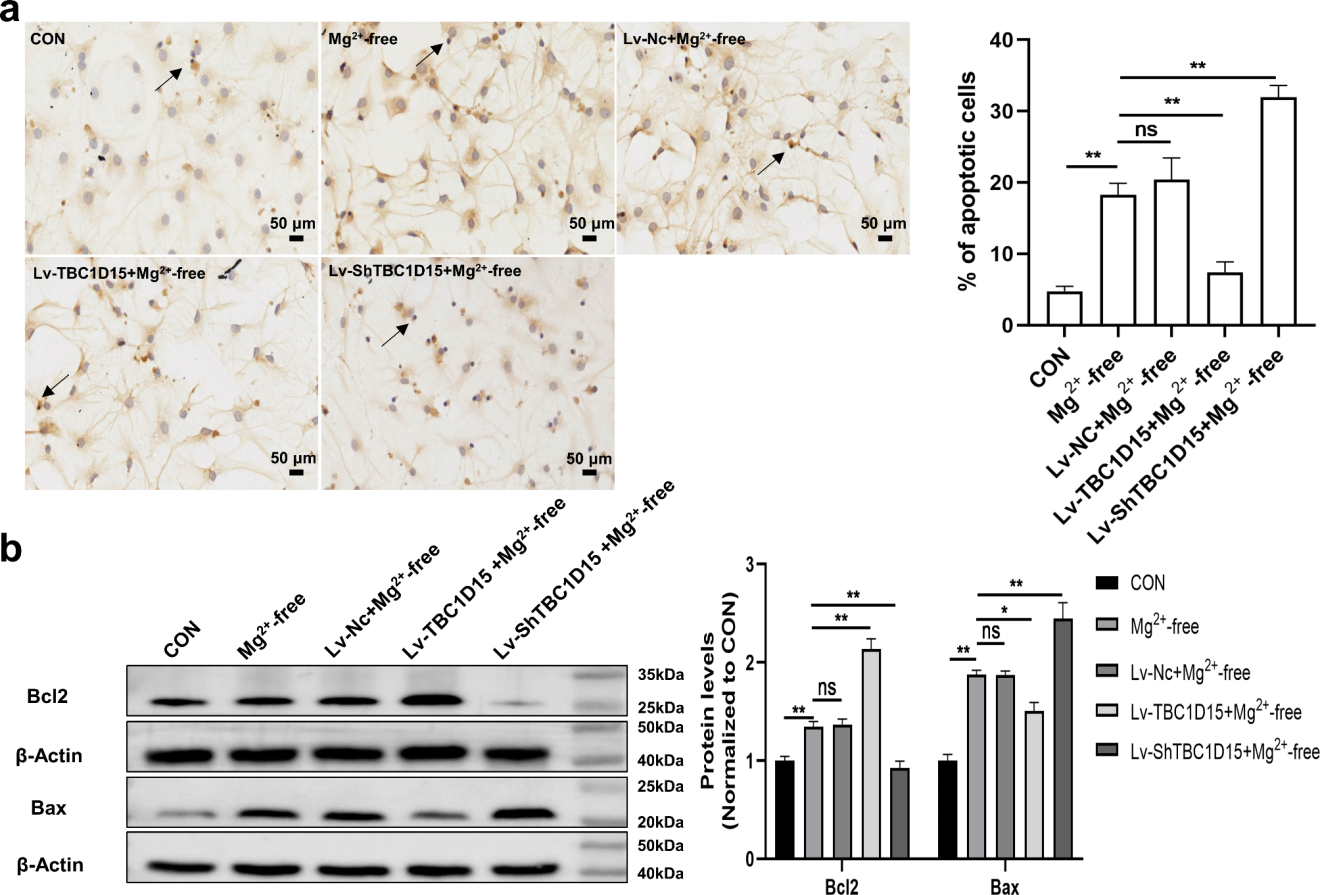
Biological effect of TBC1D15 on the apoptosis of primary hippocampal neurons. (**a**) The left panel showed representative TUNEL staining (magnification, ×400). The right panel showed statistics analysis results of percentages of TUNEL-positive cells per field in different groups; (**b**) Changes of Bcl2 and Bax protein levels among all groups. The black arrows indicate TUNEL-positive cells. ns: not significant, *P* > 0.05; * *P* < 0.05, ** *P* < 0.01, *n* = 5.

Apoptosis of primary hippocampal neurons was analyzed using Terminal deoxyribonucleotide transferase-mediated nick-end labeling (TUNEL) staining. The results showed an increase in TUNEL-positive cells in cultured neurons after epileptiform discharge injury, compared with the CON group (Fig. 6a). TBC1D15 overexpression attenuated the number of TUNEL-positive primary hippocampal neurons, which was markedly elevated following TBC1D15 downregulation, compared with that in the Mg^2+^-free group (Fig. 6a). Subsequently, we examined the expression levels of the apoptosis-associated proteins, Bcl2 and Bax. Statistical analysis of the WB results demonstrated that compared with the control primary hippocampal neurons, the expression of Bcl2 and Bax increased in the Mg^2+^-free group (Fig. 6b). Notably, lower Bax and higher Bcl2 levels were observed in the Lv-TBC1D15 + Mg^2+^-free group than in the Mg^2+^-free group; conversely, after lentivirus-mediated TBC1D15 knockdown, Bax levels increased and Bcl2 levels decreased compared with the Mg^2+^-free group (Fig. 6b).

### Effect of TBC1D15 on seizure behavior in the PILO-induced status epilepticus rat model

**Fig. 7:**
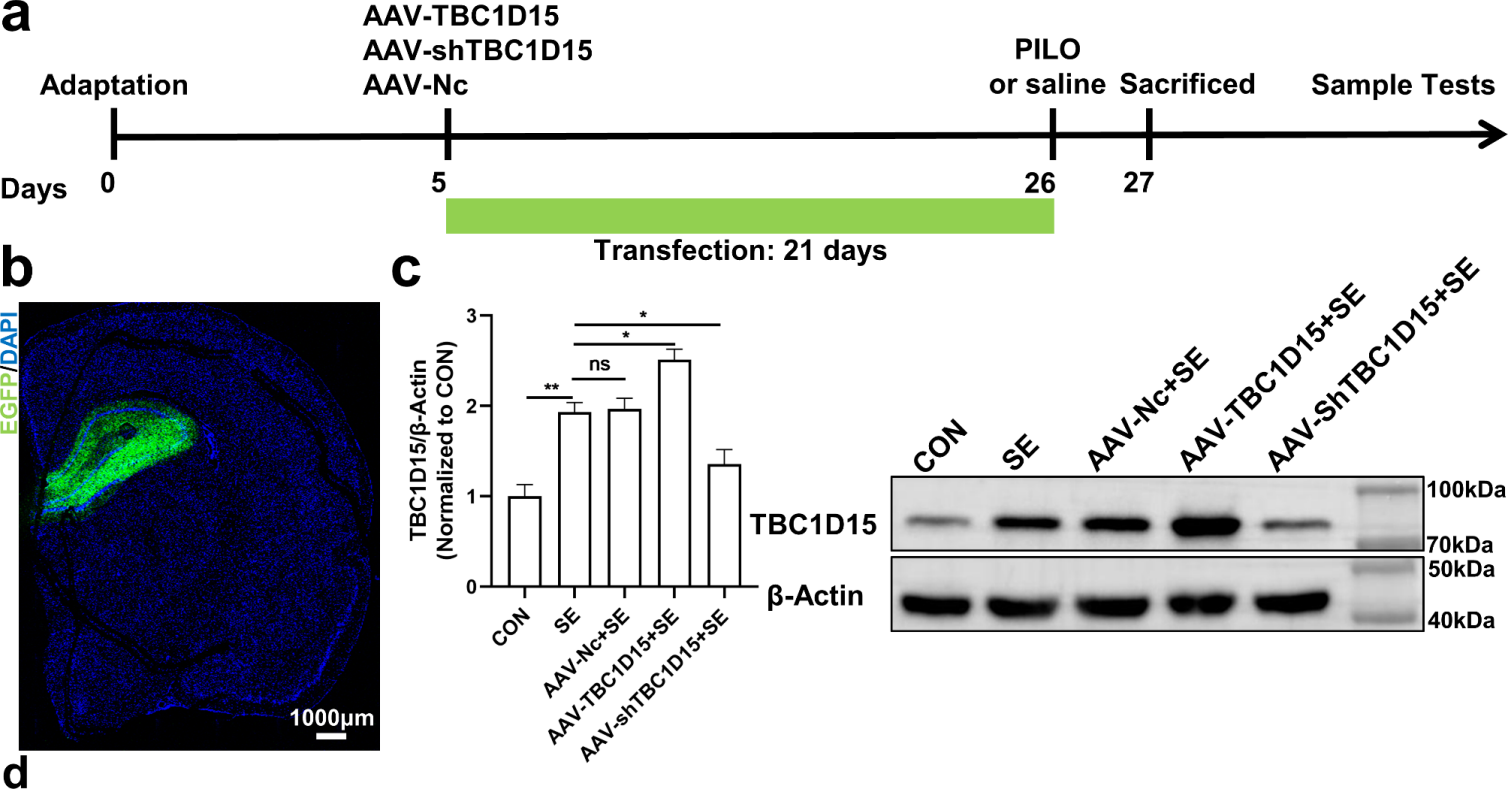
Verification of AAV infection efficacy in rat hippocampus. (**a**) Flow chart for evaluating the in vivo effect of TBC1D15 on mitochondria–lysosome membrane contact in a SE model; (**b**) Illustration of viral infusion of AAV into the hippocampus; (**c**) WB confirmed changes in protein expression of TBC1D15 in the hippocampus after injection with AAV. ns: not significant, *P* > 0.05; * *P* < 0.05, ** *P* < 0.01, *n* = 5.

Next, as depicted in the flow chart (Fig. 7a), the functional role of TBC1D15 in vivo was investigated via TBC1D15 overexpression or knockdown using an AAV-mediated gene delivery system. Fluorescent images of the brain slices demonstrated the infusion of AAV vectors into the hippocampi (Fig. 7b). The effectiveness of TBC1D15 overexpression and knockdown was confirmed using WB with or without stimulation (Fig. 7c and Supplementary Fig. S3a).

Firstly, we aimed to investigate whether modulating the expression of TBC1D15 affected seizure behavior. The seizure behavior of rats was categorized based on the Racine scale, with the modeling results for each group detailed in Supplementary Table S1. Out of 40 PILO-treated rats, 33 exhibited a generalized convulsive (grade IV or above) seizure. Overall, the success rate of modeling was 82.5%. Compared with the SE group, overexpression of TBC1D15 increased the latency to score ≥ 4 and reduced seizure duration, whereas downregulation of TBC1D15 shortened the latency to score ≥ 4 and prolonged seizure duration (Supplementary Fig. S4a and S4b). In comparison to the SE group, the AAV-Nc showed no impact on latency to score ≥ 4 and seizure duration in the PILO-induced SE rat model (Supplementary Fig. S4 a and S4b). Nevertheless, TBC1D15 had no effect on seizure severity, number of seizure, and number of seizure (score ≥ 4) (Supplementary Fig. S4c–S4e).

### Effect of TBC1D15 on mitochondria–lysosome membrane contact in the PILO-induced status epilepticus rat model

**Fig. 8:**
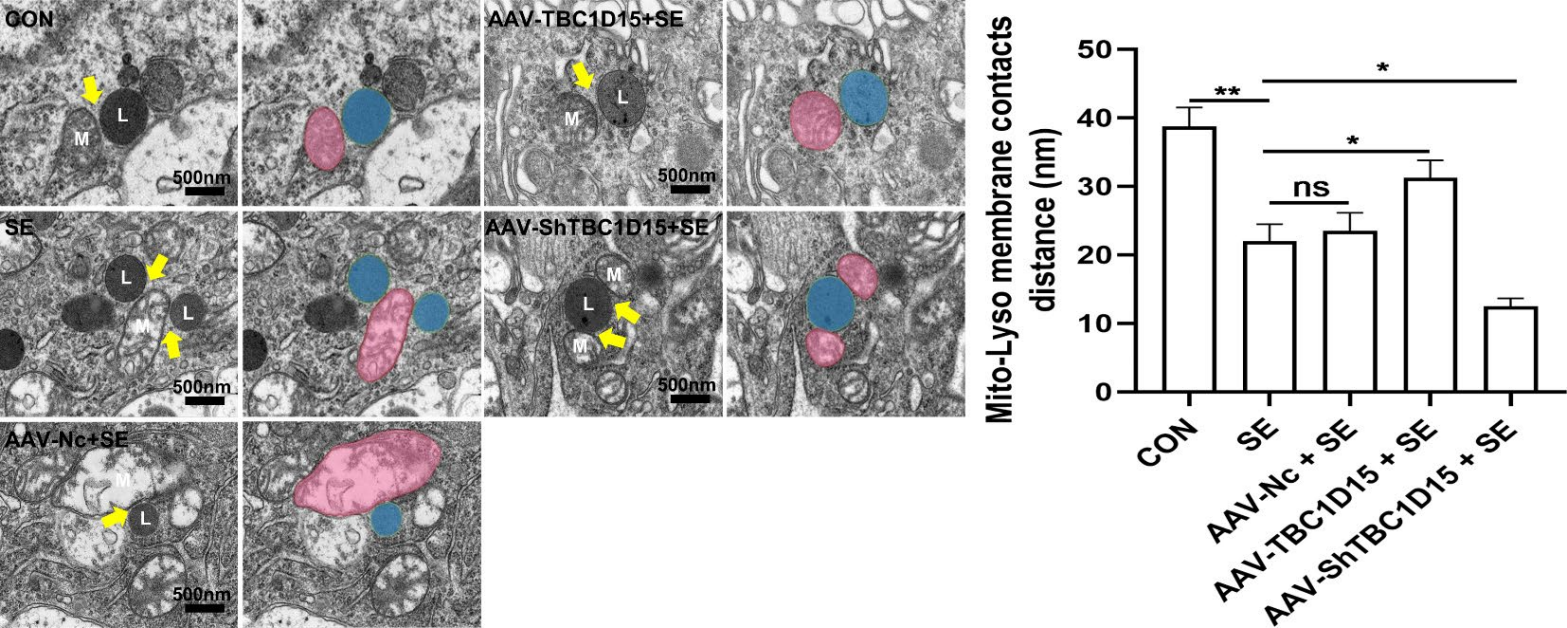
Influence of TBC1D15 on mitochondria–lysosome membrane contact in an in vivo SE rat model. The left panel showed representative TEM images of mitochondria–lysosome membrane contact (yellow arrows) in neurons in the CA3 region of the hippocampi (magnification, ×12k). The right panel showed the corresponding statistics analysis results. ns: not significant, *P* > 0.05; * *P* < 0.05, ** *P* < 0.01, *n* = 5.

We further investigated the effect of TBC1D15 on mitochondria–lysosome membrane contact in an in vivo model. Transmission electron microscopy (TEM) showed changes in the mitochondria–lysosome membrane contact in the CA3 region of the hippocampi (Fig. 8). Neurons in the SE group exhibited shorter contact distances than those in the CON group (Fig. 8). These results suggest that SE challenge tightly tethered the association between mitochondria and lysosomes in the hippocampi, the effect of which was abolished by TBC1D15 overexpression and exacerbated by TBC1D15 knockdown following SE damage (Fig. 8).

### TBC1D15 exerts neuroprotective effects via regulation of mitochondria–lysosome membrane contact in the PILO-induced status epilepticus rat model.

**Fig. 9:**
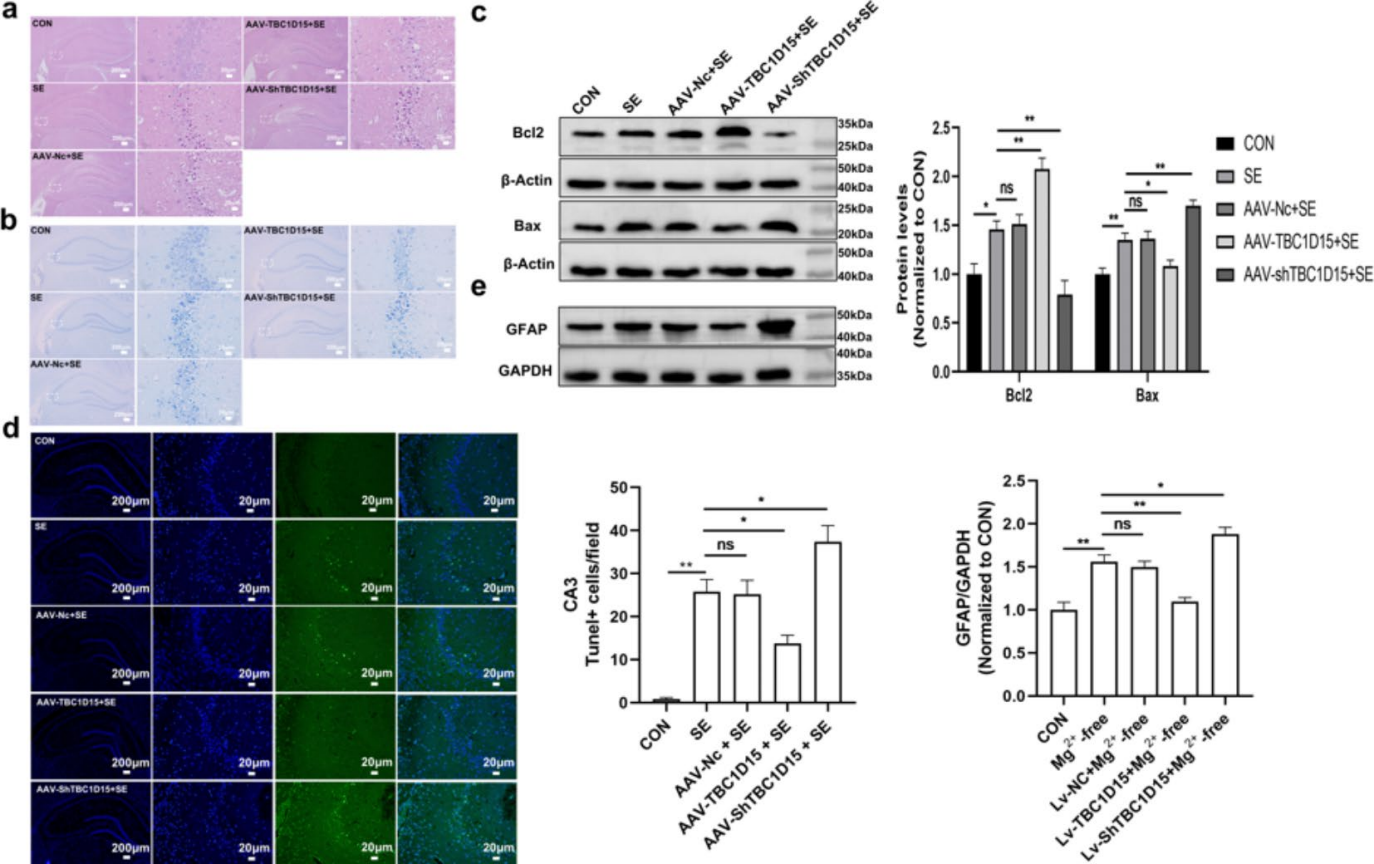
Impact of TBC1D15 on neuronal damages and GFAP expression in the hippocampus. (**a** and **b**) HE and Nissl staining of the CA3 region in the hippocampus from different groups; (**c**) Levels of Bcl2 and Bax in different groups; (**d**) The left panel showed representative TUNEL staining in CA3 areas of the hippocampus (magnification, ×40 and ×200); (**e**) GFAP expression in various group. ns: not significant, *P* > 0.05; * *P* < 0.05, ** *P* < 0.01, *n* = 5.

First, to investigate the pathological changes in SE rat hippocampi, the CA3 regions were evaluated using hematoxylin and eosin (HE) and Nissl staining. Increased solidified and atrophied neurons were observed in the CA3 regions of the rat hippocampi after SE 24 h, compared with the CON group (Fig. 9a). TBC1D15 overexpression alleviated the increase in damage to neurons caused by SE, whereas suppression of TBC1D15 dramatically aggravated SE-induced hippocampal neuron injury in the CA3 area (Fig. 9a). These pathological changes were confirmed using Nissl staining (Fig. 9b). Notably, alterations in TBC1D15 expression levels did not affect neurons in the hippocampal CA3 regions prior to stimulation (Supplementary Fig. S3b and S3c).

We tested the effect of TBC1D15 on the expression levels of apoptosis-related proteins in the hippocampi of SE rats. As shown in ​Fig. 9c, TBC1D15 overexpression suppressed Bax and induced Bcl2 expression, whereas TBC1D15 downregulation increased Bax and decreased Bcl2 protein expression, compared with the SE group. Apoptosis was confirmed using TUNEL staining (Fig. 9d). Additionally, the impact of TBC1D15 on hippocampal astrogliosis was assessed by measuring GFAP expression. The results indicated that PILO treatment elevated GFAP expression levels compared with the CON group. Furthermore, TBC1D15 knockdown resulted in higher GFAP levels compared with the SE group, whereas TBC1D15 overexpression decreased GFAP levels (Fig. 9e).

## Discussion

**Fig. 10:**
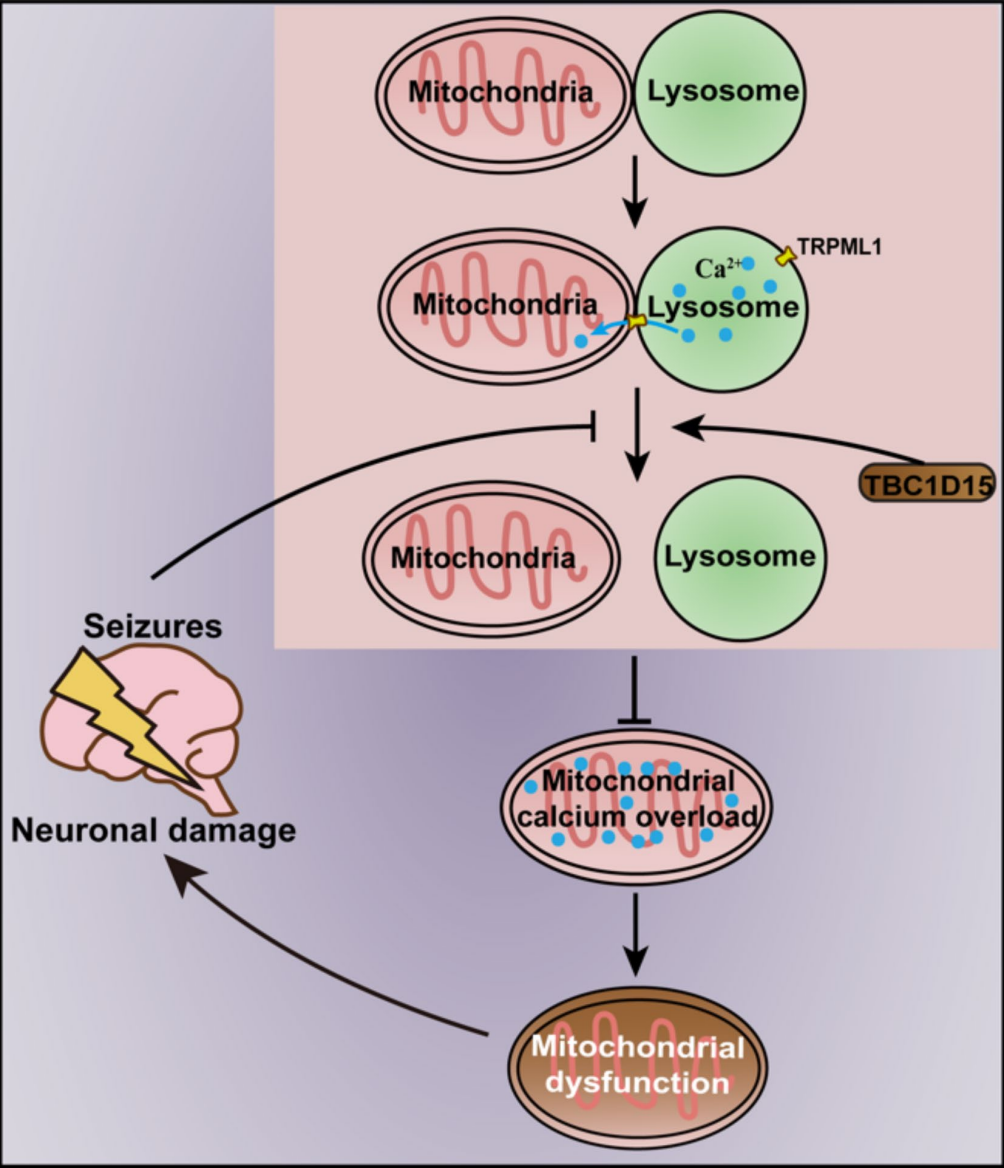
Scheme of TBC1D15-exerted neuroprotection against seizure-induced neuronal damage. The membranes of mitochondria and lysosomes can form dynamic contact under physiological conditions, and the membranes in contact site provide a platform for calcium release from the lysosome to the mitochondria. TBC1D15 promotes untethering of mitochondria–lysosome membrane contacts, which is critical for regulating organelle network dynamics and maintaining cellular homeostasis. Seizures induce abnormalities in TBC1D15-regulated mitochondria–lysosome membrane contact, resulting in mitochondrial calcium overload, which is responsible for mitochondrial dysfunction and exacerbates neuronal damage.

Epilepsy is a prevalent chronic neurological disorder; however, its pathogenesis remains unclear^19^. New antiepileptic drugs targeting the central neural mechanisms have failed to reduce the proportion of patients with drug-resistant epilepsy^20^. Consequently, further exploration of the cellular and molecular mechanisms underlying epilepsy is required to provide novel insights into treatment. The results of our study indicate that TBC1D15 exerts neuroprotective effects in both in vivo SE and in vitro epileptiform discharge models. Seizures result in abnormal mitochondria–lysosome membrane contact, as manifested by longer contact duration and shorter spatial contact distance. This may lead to an excessive influx of calcium into the mitochondria from lysosomes and subsequent mitochondrial calcium overload. Consequently, dysfunctional mitochondria and ROS accumulation ultimately cause neuronal injury. Nevertheless, TBC1D15 overexpression promoted untethering of mitochondria–lysosome membrane contacts, thus preventing mitochondrial calcium overload and restoring mitochondrial function. Accordingly, TBC1D15 was thought to protect mitochondrial function by regulating mitochondria–lysosome membrane contact and alleviating mitochondrial calcium overload, resulting in preserved mitochondrial function and relieved neuronal injury in the face of seizure challenge, as summarized in the schematic diagram shown in Fig. 10. To the best of our knowledge, this is the first study to explore the role of TBC1D15 in seizure and describe the relationship between mitochondria–lysosome membrane contact and mitochondrial calcium in epileptiform discharge settings.

The key finding of this study was that TBC1D15 confers neuroprotection against seizure-induced neuronal injury by bridging mitochondria–lysosome membrane contact and mitochondrial calcium homeostasis. TBC1D15 is a TBC domain-containing protein that functions as a GTPase-activating Rab7 GTPase. TBC1D15 reportedly participates in multiple cellular processes such as mitochondria–lysosome membrane contact^16^, mitochondrial networks^21^, lysosomal regeneration^22,23^, intracellular substance transport^24^, and immune responses^25^. Additionally, TBC1D15 has a marked effect on cardiovascular diseases and may be a novel therapeutic target for acute myocardial infarction^26,27^. However, little is known regarding the role of TBC1D15 in the brain. Research has indicated that SE can result in hippocampal damage, including astrogliosis and neuronal loss^28,29^. These pathophysiological events increase the risk of subsequent seizures (i.e., epileptogenesis). In our study, we found that TBC1D15 colocalized with neurons in the rat hippocampus. In the SE rat model, overexpression of TBC1D15 prevented hippocampal neuronal injury, apoptosis, and astrogliosis, which may explain the prolonged latency of seizure onset and shortened seizure duration in rats with TBC1D15 overexpression. Abundant evidence has indicated that seizures can damage mitochondria, resulting in a decreased MMP, increased mitochondrial permeability, and the release of cytochrome c and apoptosis-inducing factors into the cytoplasm^2,30^. This process induced apoptosis and heightens susceptibility to epileptic damage. Therefore, mitochondrial dysfunction is not only a consequence of seizures but may also directly contribute to epileptogenesis. This correlates well with our results, which showed altered expression of Bax and Bcl2, proteins that control mitochondria-mediated apoptosis pathway, along with reduced MMP, elevated ROS levels, decreased cell viability, and destruction of the hippocampal structure during epileptiform discharge or SE insult. Furthermore, our study highlights the fact that TBC1D15 exerts a neuroprotective effect by mitigating mitochondrial dysfunction and ROS accumulation induced by epileptiform discharge, which is consistent with the findings of Yu et al. in an acute myocardial infarction model^26^.

One of the pivotal functions of mitochondria is to buffer cytosolic calcium and regulate intracellular calcium homeostasis. Excessive calcium ion influx into neurons has been observed in rats subjected to SE induced by bicuculline and L-allylglycine^31^. When the cytosolic calcium concentration exceeds the mitochondrial calcium buffering capacity, mitochondrial calcium overload can occur, which is responsible for mitochondrial swelling, activation and release of mitochondrial proteins that trigger cell death, and loss of the outer mitochondrial membrane integrity^1^. Mitochondrial calcium overload has been shown to cause cell death, particularly in the CA3 regions, which are highly sensitive to seizure-induced cell death in the central nervous system^1,31–33^. Mitochondria are the primary site for calcium accumulation during prolonged seizures^32^. Consequently, mitochondrial calcium homeostasis plays a crucial role in the development of epilepsy. Experiments in the Mg^2+^-free induced-epileptiform discharge model of primary hippocampal neurons have shown excessive mitochondrial calcium accumulation, which in turn determines mitochondrial dysfunction and neuronal apoptosis. Correspondingly, disruptions in the cellular structure of the CA3 region are evident, accompanied by a notable increase in apoptotic cells in the PILO-induced SE model. Overexpression of TBC1D15 alleviated mitochondrial calcium overload, restored the MMP, prevented excess intracellular ROS accumulation, increased Bcl2 expression, decreased Bax expression, and rescued histopathological changes in the CA3 region.

Recently, mitochondria-lysosome membrane contact has been identified as a crucial pathway mediating crosstalk between these two organelles in neurons^34^. Moreover, recent research has linked the dynamics and function impairment of mitochondria-lysosome membrane contact to various neurodegenerative diseases^34–36^. Importantly, mitochondria–lysosome membrane contact allows for bidirectional regulation between these organelles, as well as the mechanistic regulation of organelle network dynamics, function, and metabolite transfer. Results from a recent study have suggested that mitochondria-lysosomal contacts regulate mitochondrial calcium dynamics via lysosomal calcium efflux channels, TRPML1 and TBC1D15 knockout induced-prolonged mitochondria–lysosome membrane contact tethering can increase lysosomal calcium transfer into mitochondria^15^. In this study, TBC1D15 serves as a bridge between the mitochondria–lysosome membrane contact and mitochondrial calcium. Prolonged contact duration and shorter contact distances were observed in hippocampal neurons in both in vivo SE and in vitro epileptiform discharge models, which was alleviated by TBC1D15 overexpression and exacerbated by TBC1D15 knockdown, suggesting that TBC1D15 regulates this process. Moreover, we found that the suppression of TBC1D15 aggravated mitochondrial calcium overload. However, this phenomenon could be reversed by TBC1D15 knockdown combined with TRPML1 antagonist ML-SI3 treatment, which indirectly indicated that epileptiform discharge-induced abnormal mitochondria–lysosome membrane contact might result in an excessive influx of calcium into mitochondria from lysosomes, leading to mitochondrial calcium overload. Thus, this study provides evidence that membrane contact can provide a platform for component exchange between the two organelles. The misregulation of substance exchange can result in substance accumulation and mitochondrial dysfunction. However, further studies are needed to explore the specific mechanism of TBC1D15-regulated mitochondria–lysosome membrane contact and mitochondrial calcium overload in epilepsy.

Overall, TBC1D15-regulated mitochondria–lysosome membrane contact exerts neuroprotective effects by alleviating mitochondrial calcium overload in seizure. This study identified a novel therapeutic target for the clinical treatment of seizure. These findings also lend several implications for the dynamic interaction between two organelles and the understanding of the disease pathogenesis. Although our findings shed new light on TBC1D15’s function in mitochondrial calcium, further studies are still warranted to confirm the role of TBC1D15 in the maintenance of mitochondrial homeostasis, especially mitochondrial calcium homeostasis.

## Methods

### Animals and grouping

Male Sprague–Dawley (SD) rats (5–6 weeks old) weighing 160–180 g were obtained from the Laboratory Animal Center of Henan Province (Zhengzhou, China) and the rats were randomly divided into five groups: CON (no infection), SE, (no infection), AAV-Nc + SE (infected with negative control AAV containing EGFP), AAV-TBC1D15 + SE (infected with TBC1D15 overexpression AAV), and AAV-shTBC1D15 + SE (infected with TBC1D15 knockdown AAV) groups. All rats were maintained under standard conditions with a 12-h light/dark cycle and ad libitum access to sterilized food and water. Experimental procedures were approved by the Animal Ethics Committee of Zhengzhou University (No. 2023-YYY-021) in accordance with the National Institutes of Health Guide for the Care and Use of Laboratory Animals and ARRIVE guidelines.

### Stereotaxic injection of adeno-associated virus (AAV)

TBC1D15 overexpression (GV467, CMV-betaGlobin-MCS-EGFP-3Flag-SV40 PolyA), knockdown (GV478, U6-MCS-CAG-EGFP), and negative control (GV478, U6-MCS-CAG-EGFP) AAV9 (titer = 3E + 12 v.g./ml) were generated by Genechem Co., Ltd. (Shanghai, China). Supplementary Table S2 provides the list of shRNA sequences targeting TBC1D15. After 5 days of adaptation, the rats were anesthetized with pentobarbital (70 mg/kg, intraperitoneally) and immobilized on a stereotaxic frame. AAV (2.0 µl) was bilaterally microinjected into the dorsal hippocampi (anteroposterior: 3.2 mm, mediolateral: ±2.4 mm, and dorsoventral: − 3.6 mm) with an injection rate of 0.2 µl/min using a micro syringe (Hamilton, USA). Three weeks after injection, the infection efficiency was verified using EGFP fluorescence imaging and WB.

### In vivo model of status epilepticus (SE) group

The PILO-induced SE model was established 21 days after the AAV injection. To induce SE, rats in the SE, AAV-Nc + SE, AAV-TBC1D15 + SE, and AAV-shTBC1D15 + SE groups were intraperitoneally injected with PILO (35 mg/kg, intraperitoneally) 18–20 h after pretreatment with lithium chloride (128 mg/kg, intraperitoneally). To prevent peripheral cholinergic symptoms, methyl-scopolamine (1 mg/kg) was subcutaneously injected 30 min before PILO administration. Successful SE models were considered when the rats’ seizure behavior reached grade IV and above without returning to normal behavioral patterns, according to the Racine scale: 0-normal behavior, I-mouth and facial cramps, II-head nodding, III-forelimb clonus, IV-rearing, and V-rearing and falling^37^. One hour after the onset of SE, seizures were stopped with diazepam (10 mg/kg, intraperitoneally). The CON group received an equal volume of saline instead of PILO, and the other procedures were similar to those used for the SE animals. We measured the seizure behavior including: latency time, seizure duration, seizure severity, number of seizure, and number of seizure (score ≥ 4).

### Transmission electron microscopy (TEM)

TEM was used to observe the neuronal mitochondrial and lysosomal ultrastructure in the CA3 region of the hippocampi. Samples were fixed in 2.5% glutaraldehyde and then post-fixed in 1% osmium tetroxide. After dehydration with a series of ethanol (30%, 50%, 70%, 80%, 95% and 100%) and acetone solutions, the tissues were embedded in epoxy resin. Resin blocks were cut using an ultramicrotome (Leica, Germany) equipped with a diamond slicer and fixed onto the 150-mesh copper grids. The ultrathin Sect. (70 nm thickness) were stained with 2% uranium acetate and 2.6% lead citrate and observed using a 20–120 kV transmission electron microscope (Hitachi HT7800 Electron Microscope, Hitachi, Japan). The distance of mitochondria–lysosome membrane contact was calculated using the ImageJ software (v.1.48, http://imagej.nih.gov/ij/).

### Histology

After the anesthetized rats were transcardially perfused with PBS, followed by 4% paraformaldehyde, brain tissues were harvested and postfixed in 4% paraformaldehyde. Subsequently, the tissue specimens were embedded in paraffin. Paraffin sections of 4-µm thickness were sliced. To evaluate neuronal damage in the hippocampal CA3 region, HE and Nissl staining were performed following the manufacturer’s protocol. TUNEL staining was performed to determine neuronal apoptosis in the hippocampal CA3 area of the rats using an in situ cell death detection kit (11684817910, Roche Diagnostics, Switzerland). Briefly, the paraffin-embedded tissue sections were dewaxed, hydrated, and incubated with a proteinase K solution for 20 min at 37 °C. Afterwards, the sections were incubated with the TUNEL reaction mixture for 1 h at 37 °C in a humidified chamber. Finally, the DAPI-containing anti-fluorescence quencher was used to seal the glass sections. The staining results were examined via microscopy.

### Immunofluorescence

Fresh brain tissues were embedded in OCT and then sliced into 10 μm coronal sections with a Leica cryostat. Frozen sections were washed in PBS, permeabilized with 0.25% Triton X-100, and blocked with 5% goat serum for 30 min. Subsequently, the sections were incubated with rabbit anti-TBC1D15 (1:20, ab121396, abcam, UK) and mouse anti-NeuN (1:200, ab104224, abcam, UK) overnight at 4 °C. The next day, the sections were incubated with CoraLite594-conjugated Goat Anti-Rabbit IgG(H + L) (1:200, SA00013-4, Proteintech, China) and CoraLite488-conjugated Goat Anti-Mouse IgG(H + L) (1:200, SA00013-1, Proteintech, China) for 1 h at 37 °C. After washing with PBS, the tissue sections were sealed with an anti-fluorescence attenuation sealant containing DAPI. Pictures were taken by a fluorescence microscope (Olympus, Japan). To verify the AAV infection efficiency, frozen sections sealed with DAPI-containing anti-fluorescence quencher were observed under a Zeiss LSM 880 confocal microscope (Carl Zeiss).

### Primary hippocampal neuron culture

The cerebral hippocampus was prepared from neonatal SD rats (within 24 h). Tissues were minced and digested with 0.25% trypsin (03-050-1ACS, Biological Industries, Israel) and DNase at 37 °C for 15 min, followed by centrifugation at 1000 rpm for 5 min. After discarding the supernatant, cell suspensions with a density of 3.0 × 10^5^ cells/mL were prepared in Dulbecco’s Modified Eagle Medium (DMEM, 01-052-1ACS, Biological Industries, Israel) containing 10% FBS (04-002-1B, Biological Industries, Israel), 1% penicillin/streptomycin (03-031-1B, Biological Industries, Israel), and 1% glutamine (03-020-1B, Biological Industries, Israel), and then seeded in 0.1 mg/mL poly-d-lysine-coated 6-well plates. The DMEM was replaced 4–6 h later with a maintenance medium consisting of 96% neurobasal-A (10888022, Invitrogen, USA), 2% B27 supplement (17504044, Invitrogen, USA), 1% penicillin-streptomycin, and 1% glutamine. Half of the culture medium was changed every 3 days. Neuronal morphology was observed using an inverted microscope (Olympus, Japan).

### Primary hippocampal neuron grouping and lentiviral infection

The lentivirus vectors (titer = 2E + 9 TU/ml) for TBC1D15 overexpression, TBC1D15 shRNA, and gcGFP-empty control lentivirus vectors were purchased from Genechem Co., Ltd. (Shanghai, China) to infect the primary hippocampal neurons. For overexpression, rat full-length cDNA of TBC1D15 was cloned into a Ubi-MCS-3FLAG-CBh-gcGFP-IRES-puromycin lentiviral vector (GV492, Genechem, Shanghai, China). To achieve knockdown, short hairpin RNA (shRNA) of rat TBC1D15 was cloned into a hU6-MCS-CBh-gcGFP-IRES-puromycin lentiviral vector (GV493, Genechem, Shanghai, China). Supplementary Table S2 provides the list of shRNA sequences targeting TBC1D15. On day 6, the primary hippocampal neurons were randomly divided into five groups: CON, Mg^2+^-free, Lv-Nc + Mg^2+^-free, Lv-TBC1D15 + Mg^2+^-free, and Lv-shTBC1D15 + Mg^2+^-free groups. The latter three groups were infected with empty vectors, TBC1D15 overexpression lentiviruses, or shRNA-TBC1D15 lentivirus with HiTransG A infection-enhancing solution according to the multiplicity of infection (MOI, MOI = 10). After 12 h, the culture medium containing the Lv particles was removed and replaced with a fresh maintenance medium. Three days later, gcGFP fluorescence was observed under fluorescent microscopy to evaluate the infection efficiency. Changes in TBC1D15 expression were further verified by WB.

### Neurite length measurement

To measure the length of neurites, 20 neurons were randomly chosen, and their images were obtained using fluorescence or brightfield microscopy. The neurite length was measured using ImageJ software (v.1.48, http://imagej.nih.gov/ij/).

### In vitro model of epileptiform discharges

After 72 h of infection, the maintenance medium of the Mg^2+^-free, Lv-Nc + Mg^2+^-free, Lv-TBC1D15 + Mg^2+^-free, and Lv-shTBC1D15 + Mg^2+^-free groups was replaced with Mg^2+^-free extracellular fluid (2.5 mM KCl, 145 mM NaCl, 10 mM glucose, 0.001 mM glycine, 2 mM CaCl_2_, 10 mM HEPES, 1mM MgCl_2_, pH 7.3) to establish a hippocampal neuron epileptiform discharge model, as previously described^38,39^. The CON group cultures were treated with normal extracellular fluid with 1 mM MgCl_2_ at the same time. After 3 h of culture, the extracellular fluid with or without MgCl_2_ was removed and replaced with a fresh maintenance medium.

### Assessment of mitochondrial membrane potential (MMP)

The MMP of the primary hippocampal neurons was measured using TMRE staining (C2001S, Beyotime Biotechnology, China). Briefly, cells were incubated with 1X TMRE working solution for 30 min at 37 °C followed by PBS washing, and subsequently visualized using a fluorescence microscope (Olympus, Japan). ImageJ software was used to analyze the mean fluorescent intensity.

### Intracellular reactive oxygen species (ROS) detection

DHE dye (S0063, Beyotime Biotechnology, China) was used to detect intracellular ROS. Briefly, the primary hippocampal neurons were incubated with 3.5 µM DHE and 1 µM *Hoechst* for 30 min at 37 °C in the dark and washed with PBS. Micrographs were obtained using a fluorescence microscope (Olympus, Japan). The mean fluorescence intensity was measured by ImageJ software (v.1.48, http://imagej.nih.gov/ij/).

### Mitochondrial calcium imaging

For mitochondrial calcium imaging assays, on day 6, the primary hippocampal neurons were randomly divided into six groups: control (CON group, no infection), Mg^2+^-free (no infection, treated with Mg^2+^-free extracellular fluid), Lv-Nc (infected with empty vectors, treated with Mg^2+^-free extracellular fluid), Lv-shTBC1D15 (infected with TBC1D15 shRNA lentiviruses, treated with Mg^2+^-free extracellular fluid), Lv-shTBC1D15 + DMSO (infected with TBC1D15 shRNA lentiviruses, treated with Mg^2+^-free extracellular fluid), and Lv-shTBC1D15 + ML-SI3 (infected with TBC1D15 shRNA lentiviruses, treated with Mg^2+^-free extracellular fluid) groups. Lentiviral infection was performed in the same manner. After 3 days, the Lv-shTBC1D15 + DMSO and Lv-shTBC1D15 + ML-SI3 groups were pretreated with a maintenance medium containing DMSO or ML-SI3 (30 mM) for 30 min before the in vitro model of epileptiform discharge construction. Mitochondrial calcium levels were measured using Rhod-2 AM (40776ES50, Yeasen, China). After washing with Hanks buffered saline solution without calcium and magnesium (HBSS-), the cells were stained with 5 µM Rhod-2 AM at 37 °C for 30 min and then washed again with HBSS- according to the manufacturer’s instruction. Finally, mitochondrial calcium images were captured using a fluorescence microscope. The mean fluorescence intensity was analyzed by ImageJ software (v.1.48, http://imagej.nih.gov/ij/).

### Live cell time-lapse imaging

Live cells were imaged using a Zeiss LSM 880 confocal microscope. For mitochondria–lysosome membrane contact, MitoTracker Deep Red (M22426, Thermo Fisher Scientific, USA) and LysoTracker Red DND-99 (L7528, Thermo Fisher Scientific, USA) were added to the culture at final concentrations of 200 nM and 70 nM for 30 min. After washing with PBS, the cells were incubated with a ProLong Live Antifade Reagent (P36975, Thermo Fisher Scientific, USA) for 2 h according to the manufacturer’s instructions before being imaged using a confocal microscope at one frame every 3 s for a total of 60 frames. We defined the contacts as a close apposition between mitochondria and lysosomes at the start of videos with a duration of more than 18 s. Any contact exceeding 60 frames was documented for 177 s.

### Detection of cell viability and cytotoxicity

We performed CCK-8 (BMU106-CN, Abbkine, China) and LDH cytotoxicity assays (C0017, Beyotime Biotechnology, China) to evaluate cell viability and cytotoxicity, respectively, according to the manufacturer’s instructions. For the CCK-8 assay, the CCK-8 reagent was added to each well, followed by incubation for 60 min, and the absorbance was measured at 450 nm using a microplate reader. For the LDH release assay, the supernatant of the primary hippocampal neurons in a 96-well plate was collected and incubated with the LDH working solution for 30 min. The absorbance of LDH was measured at 490 nm using a microplate reader.

### Analysis of cell apoptosis by TUNEL assay

TUNEL assay was conducted using an in situ cell death detection kit according to the procedure previously described^39^. Briefly, the primary hippocampal neurons on coverslips were incubated with TUNEL working solution for 1 h after fixation with 4% paraformaldehyde and permeabilization with PBS containing 0.2% Triton X-100. The primary hippocampal neurons were supplemented with converter-POD and developed with a 3,3′-diaminobenzidine mixture. Finally, after counterstaining with hematoxylin, the coverslips were observed under a microscope (Olympus, Japan).

### Western blotting

Total protein from the primary hippocampal neurons and rat hippocampal tissues was extracted using a protein isolation kit (EpiZyme, China) according to specifications of the manufacturer and denatured in a metal bath at 100 ℃ for 10 min. Samples were separated using sodium dodecyl sulfate-polyacrylamide gel electrophoresis and transferred to polyvinylidene difluoride membranes (IPVH00010, Millipore, USA). After blocking with 5% skimmed milk at room temperature for 2 h, the membranes were then incubated with the primary antibodies overnight at 4 °C, which were rabbit polyclonal antibodies against TBC1D15 (1:1000, ab121396, abcam, UK), Bcl2 (1:1000, ab196495, abcam, UK), Bax (1:1000, #2772, Cell Signaling Technology, USA), GFAP antibodies (1:1000, 16825-1-AP, Proteintech, China), and rabbit monoclonal antibodies against β-Actin (1:1000, #4970, Cell Signaling Technology, USA). The next day, the membranes were incubated with horseradish peroxidase-labeled secondary antibodies for 1 h at room temperature. Anti-mouse and anti-rabbit secondary antibodies (511103/511203, ZEN-BIOSCIENCE, China) were then used. Densitometry was performed using enhanced chemiluminescence. The raw WB images were shown in supplementary material.

### Statistical analysis

GraphPad Prism (v.8.0, https://www.graphpad.com/) was used for statistical analysis, and data are presented as mean ± SEM. The fluorescent images under the same imaging parameters and immunoblot band intensities were determined by ImageJ software (v.1.48, http://imagej.nih.gov/ij/). The experiments were repeated independently at least thrice. Statistical significance between multiple groups was compared using one-way ANOVA analysis with a post-hoc Turkey test. Two-tailed P values < 0.05 were considered statistically significant. @@

## Electronic supplementary material

Below is the link to the electronic supplementary material.

Supplementary Material 1

Supplementary Material 2

## Data Availability

All data generated or analysed during this study are included in this published article and its supplementary information files.
