# Supplementary material for: TBC1D15-regulated mitochondria–lysosome membrane contact exerts neuroprotective effects by alleviating mitochondrial calcium overload in seizure
Source: Sci Rep. 2024 Oct 10;14:23782. doi: 10.1038/s41598-024-74388-3 (PMC11467349; doi:10.1038/s41598-024-74388-3)

TBC1D15-regulated mitochondria–lysosome membrane contact exerts neuroprotective effects by alleviating mitochondrial calcium overload in seizure

Yinyin Xie[1,+], Wanwan Zhang[1,+], Tingting Peng[1], Xiaoyi Wang[2], Xiaolei Lian[1], Jiao He[1], Cui Wang[3,*] and Nanchang Xie[1,*]

[1]Department of Neurology, The First Affiliated Hospital of Zhengzhou University, Zhengzhou, 450052, China

[2]Institutes of Biological and Medical Sciences, Suzhou Medical College of Soochow University, Suzhou, 215123, China

[3]Department of Clinical Laboratory, The First Affiliated Hospital of Zhengzhou University, Key Clinical Laboratory of Henan Province, Zhengzhou, 450052, China

[*]Correspondence: Nanchang Xie xienanchang2001@163.com; Cui Wang snowy_cui@126.com

[+]These authors contributed equally to this work.

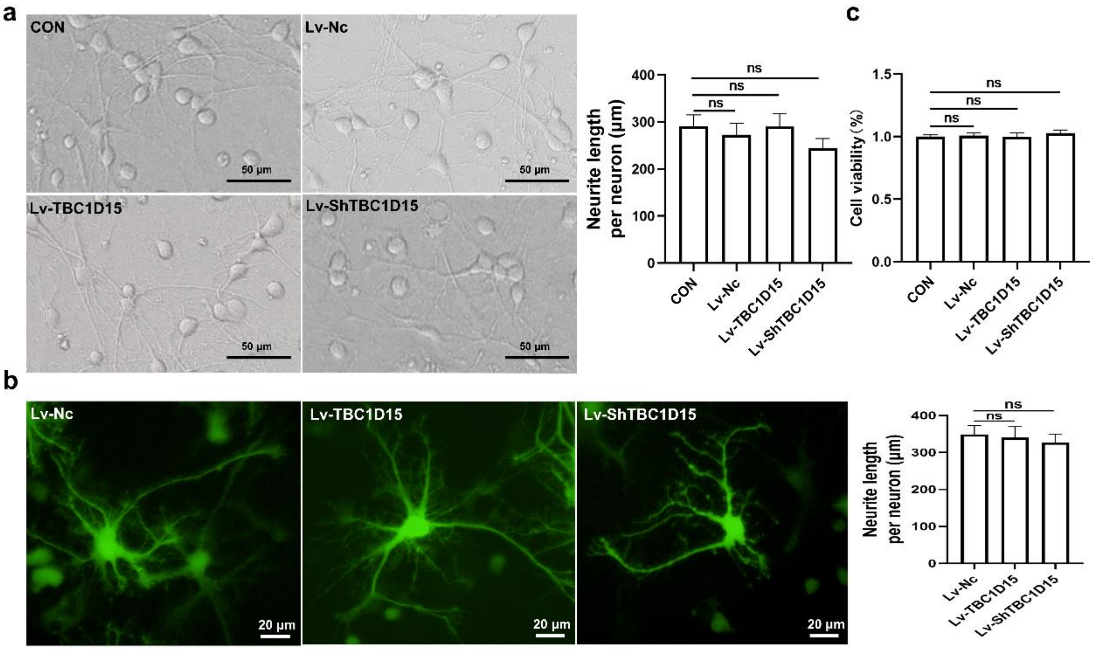

Supplementary Figure S1. Effect of lentiviral vectors on neuronal morphology and cell viability. (**a** and **b**) Left panel, representative pictures of neuronal morphology captured by brightfield and fluorescence microscopy (magnification, 200× and 400×, respectively); right panel, quantification. (**c**) The effect of TBC1D15 on the cell viability of primary hippocampal neurons. ns: not significant.

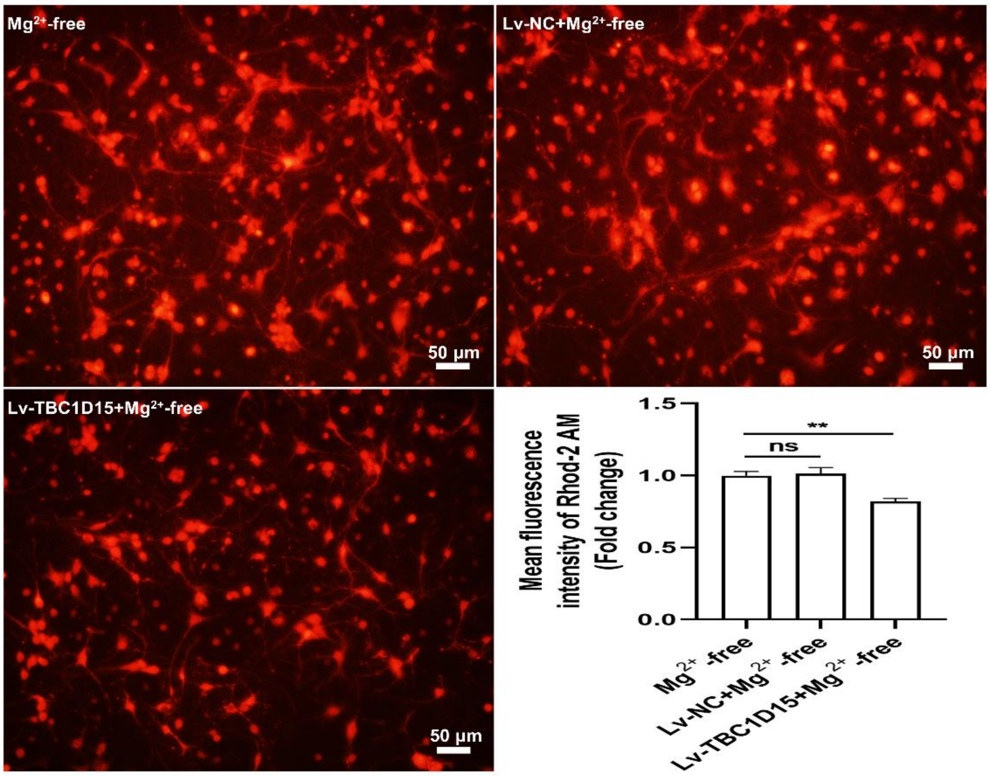

Supplementary Figure S2. The impact of TBC1D15 overexpression on mitochondrial calcium levels. Representative fluorescent images of Rhod-2 AM showed changes in mitochondrial calcium levels in each group (magnification, 200×, respectively) and the mean fluorescent intensity was quantified, n=5. ns: not significant, P >0.05; ** P < 0.01.

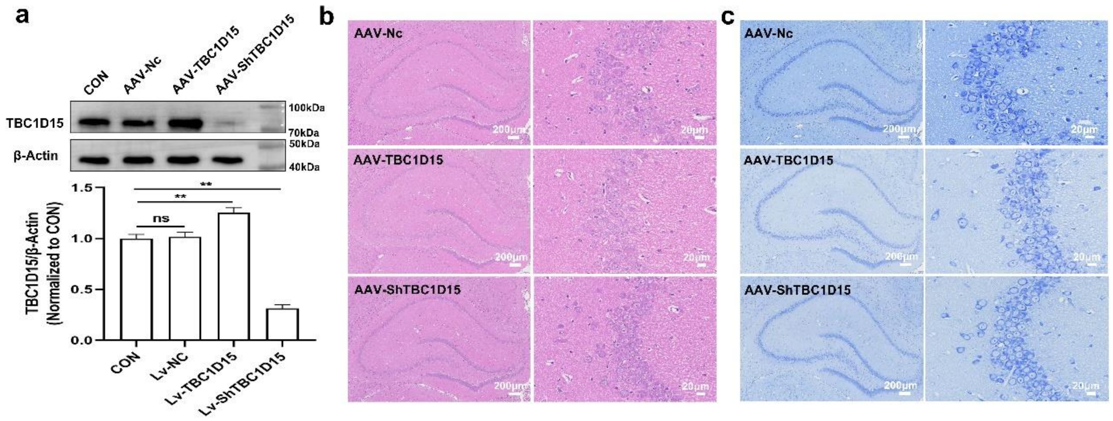

Supplementary Figure S3. Effect of altered TBC1D15 expression levels on neurons in the hippocampal CA3 regions prior to modeling. (**a**) TBC1D15 expression levels were verified by WB before modeling; (**b** and **c**) HE and Nissl staining of the CA3 region in the hippocampus from different groups (magnification, ×40 and ×200). ns: not significant, P >0.05; ** P < 0.01, n=5.

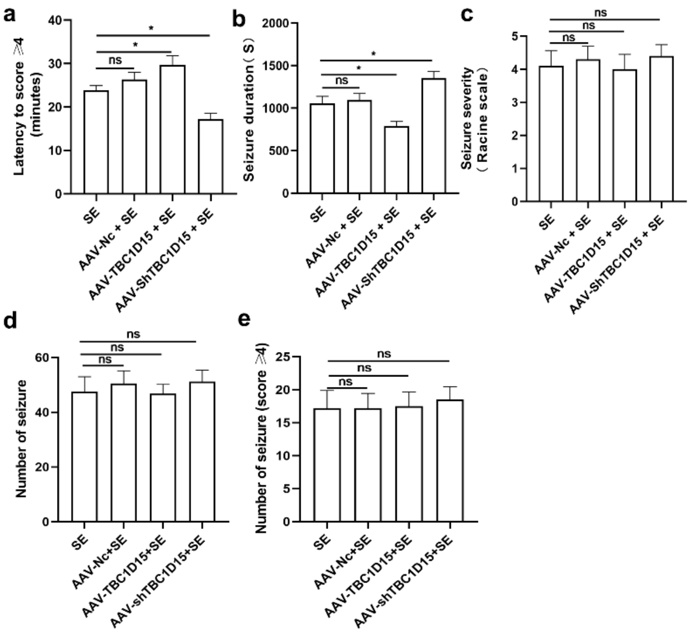

Supplementary Figure S4. Effect of TBC1D15 on seizure behavior in the pilocarpine (PILO)-induced status epilepticus rat model. (**a–e**) The statistical analysis results of latency to score ⩾ 4, seizure duration, seizure severity, number of seizure, and number of seizure (score ⩾ 4) in different groups. ns: not significant, P >0.05; * P < 0.05, n= 6 or 10.

The full gel image of the WB results in the manuscript.

Figure 1a and 1b:

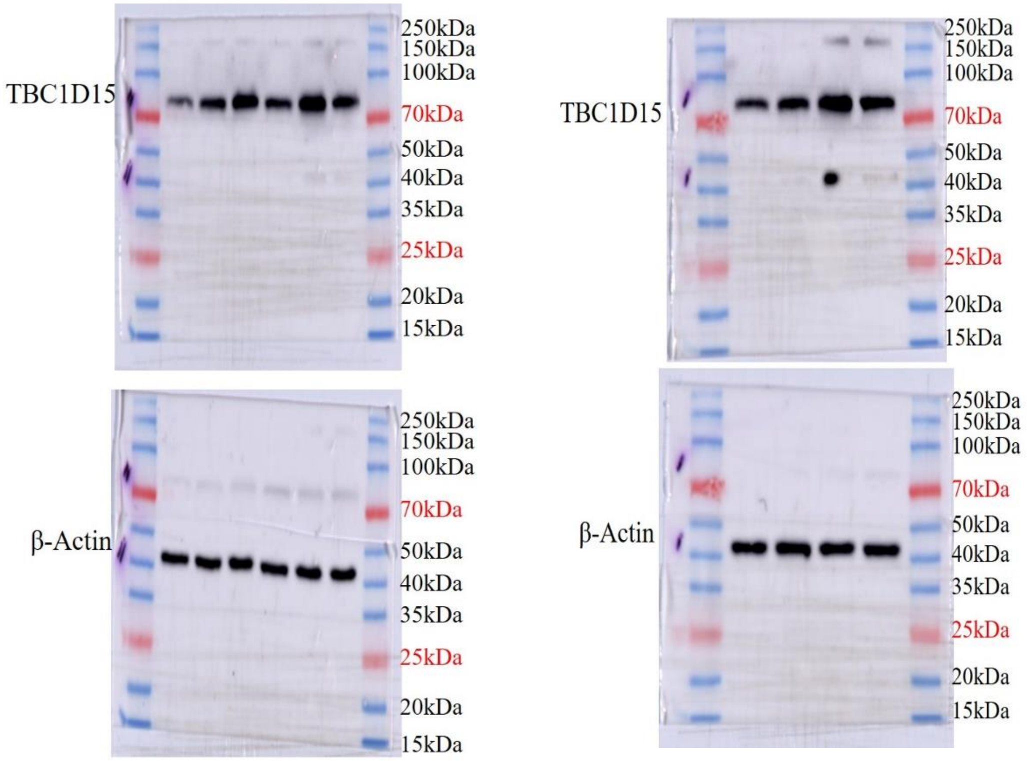

Figure 2b:

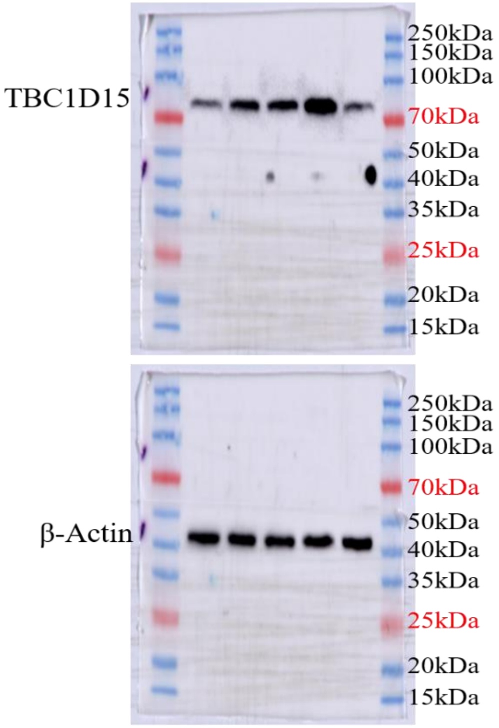

Figure 6b:

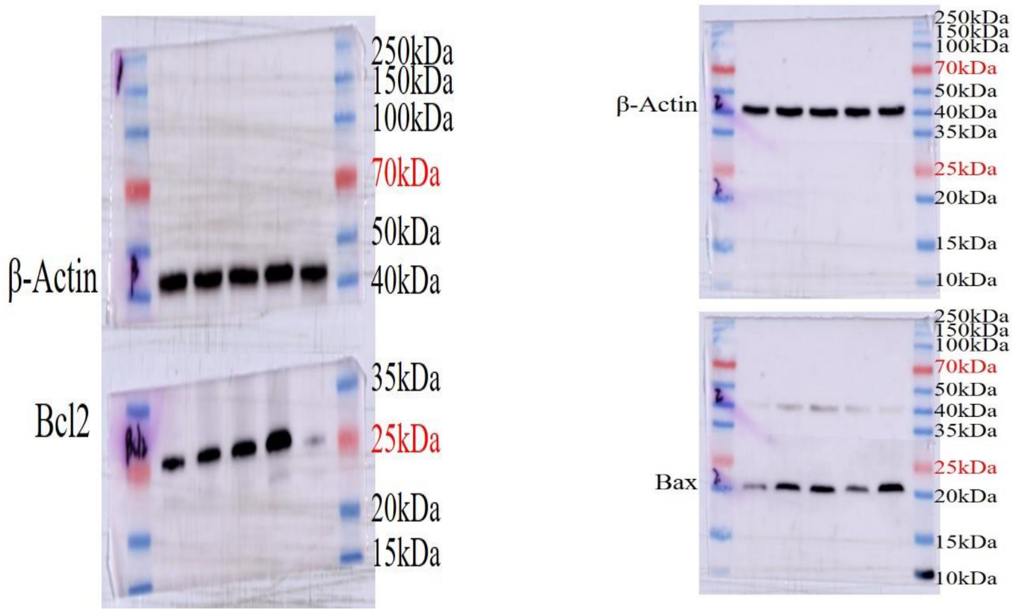

Figure 7c:

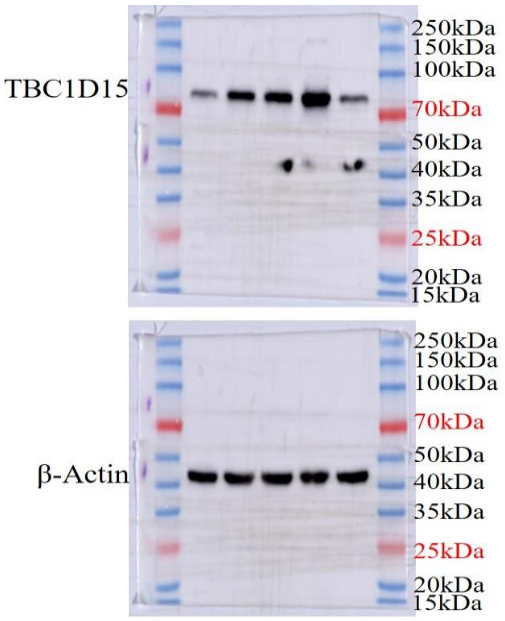

Figure 9c and 9e:

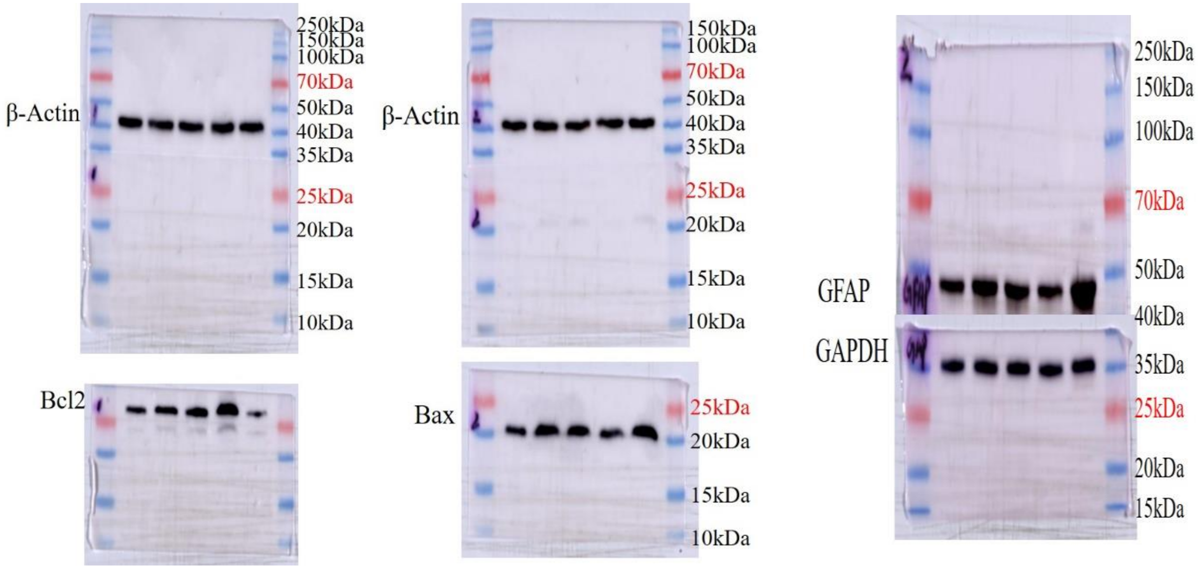

Supplementary Figure S3a:

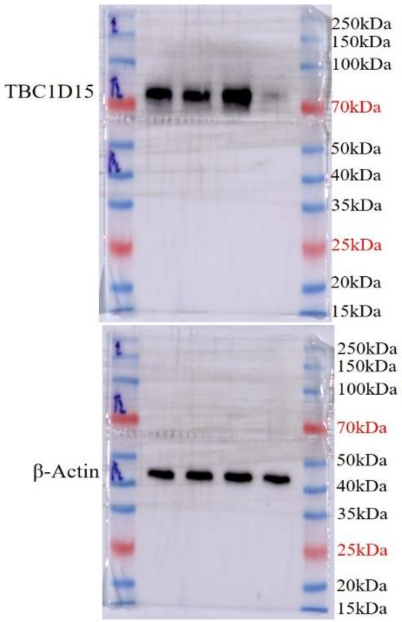

Supplement: Supplementary file 1 — Supplementary Material 1 [file 41598_2024_74388_MOESM1_ESM.pdf]
