# Supplementary material for: TBC1D15-regulated mitochondria–lysosome membrane contact exerts neuroprotective effects by alleviating mitochondrial calcium overload in seizure
Source: Sci Rep. 2024 Oct 10;14:23782. doi: 10.1038/s41598-024-74388-3 (PMC11467349; doi:10.1038/s41598-024-74388-3)
Supplement: Supplementary file 2 — Supplementary Material 2 [file 41598_2024_74388_MOESM2_ESM.pdf]

# TBC1D15-regulated mitochondria–lysosome membrane contact exerts neuroprotective effects by alleviating mitochondrial calcium overload in seizure

**Yinyin Xie[1,+], Wanwan Zhang[1,+], Tingting Peng[1], Xiaoyi Wang[2], Xiaolei Lian[1], Jiao He[1], Cui Wang[3,*] and Nanchang Xie[1,*]**

[1]Department of Neurology, The First Affiliated Hospital of Zhengzhou University, Zhengzhou, 450052, China

[2]Institutes of Biological and Medical Sciences, Suzhou Medical College of Soochow University, Suzhou, 215123, China

[3]Department of Clinical Laboratory, The First Affiliated Hospital of Zhengzhou University, Key Clinical Laboratory of Henan Province, Zhengzhou, 450052, China

[*]Correspondence: Nanchang Xie xienanchang2001@163.com; Cui Wang snowy_cui@126.com

[+]These authors contributed equally to this work.

**Supplementary Table S1** Effect of TBC1D15 on seizure score in the pilocarpine (PILO)-induced status epilepticus rat model

| **Group** | **Case (n)** | **0** | **I** | **II** | **III** | **IV** | **V** |
|---|---|---|---|---|---|---|---|
| CON | 10 | 10 | 0 | 0 | 0 | 0 | 0 |
| SE | 10 | 0 | 1 | 1 | 0 | 2 | 6 |
| AAV-Nc + SE | 10 | 0 | 1 | 0 | 0 | 3 | 6 |
| AAV-TBC1D15 + SE | 10 | 0 | 1 | 1 | 0 | 3 | 5 |
| AAV- ShTBC1D15 + SE | 10 | 0 | 0 | 1 | 1 | 1 | 7 |

**Supplementary Table S2** Sequence information of negative control and sh-TBC1D15

| **Name** | **Sequences** |
|---|---|
| Negative control (AAV) | CGCTGAGTACTTCGAAATGTC |
| Negative control (Lv) | TTCTCCGAACGTGTCACGT |
| sh-TBC1D15 #1 | GCAAGAAGAACCAGGATTTGA (effective) |
| sh-TBC1D15 #2 | GCAAGGAATGAGCGACTTACT |
| sh-TBC1D15 #3 | GCTTCAATGAAATCCTTAAGC |
